# Spinal Cord Stimulation Alleviates Chronic Peripheral Neuropathic Pain Due to Peripheral Nerve Injury: A Case Report

**DOI:** 10.7759/cureus.69383

**Published:** 2024-09-14

**Authors:** Akira Nemoto, Hisashi Date

**Affiliations:** 1 Anesthesia and Intensive Care Medicine, Akita University Graduate School of Medicine, Akita, JPN; 2 Anesthesiology, Sendai Pain Clinic Center, Sendai, JPN

**Keywords:** case report, opioid, peripheral nerve injury, peripheral neuropathic pain, spinal cord stimulation

## Abstract

Refractory neuropathic pain can be treated using opioids. We present a case of spinal cord stimulation (SCS) to ameliorate neuropathic pain and successfully stop opioid administration in a patient with median nerve injury. A woman presented with median nerve neuropathy. The intractable pain required opioids, with the dosage being gradually increased due to insufficient analgesia. The patient underwent nerve blocks and rehabilitation to reduce the opioid dosage, followed by SCS, which allowed the patient to stop opioid intake. Combined treatment modality involving nerve blocks, rehabilitation, and SCS improved the patient's pain score and quality of life.

## Introduction

Pharmacotherapy is the major treatment for neuropathic pain. Although persistent pain requires opioid agonists, they have little efficacy against neuropathic pain caused by peripheral nerve injury [1]. Opioid-induced hyperalgesia and tolerance interrupt analgesic effects. Spinal cord stimulation (SCS) is clinically beneficial for the treatment of refractory neuropathic pain. However, its efficacy against pain caused by peripheral nerve injury remains unclear. This report describes the case of a patient who required a substantial opioid dosage to manage neuropathic pain, where SCS with combined treatment modality allowed the patient to stop taking opioids.

## Case presentation

**Figure 1:**
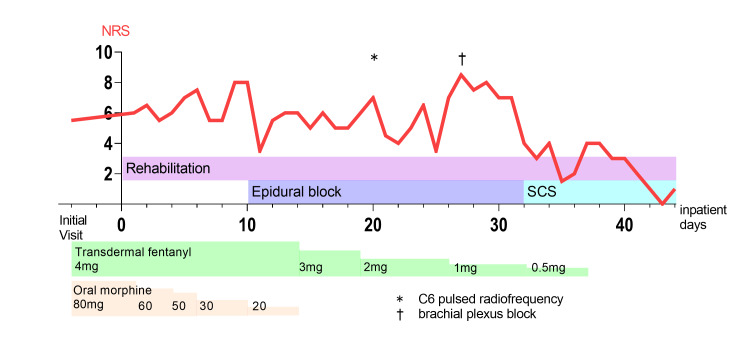
Flowchart of the inpatient treatment. *: C6 pulsed radiofrequency; †: brachial plexus block; SCS: spinal cord stimulation

A 49-year-old woman without a remarkable medical history reported pain in the right first and second upper digits. These symptoms appeared after a difficult venipuncture performed two years prior, which elicited shooting pain and hyperalgesia in the right thumb and index finger, respectively. Median nerve conduction studies did not reveal motor deficits; contrastingly, the sensory nerve showed a conduction block in the medial antebrachial cutaneous nerve. Accordingly, she was diagnosed with a right median nerve injury. The pain did not improve despite the use of nonopioid analgesic medication such as gabapentin, tricyclic antidepressants, and acetaminophen for months. Moreover, persistent hyperalgesia in the right forearm exacerbated the pain. Despite taking opioid medications, including tramadol, fentanyl patch, and morphine tablets, there was insufficient relief of the persistent pain. The opioid dosage was gradually increased to treat intractable pain for two years. However, the neuropathic pain did not improve, with 200 oral morphine mg equivalents per day being required (Figure 1).

**Figure 2:**
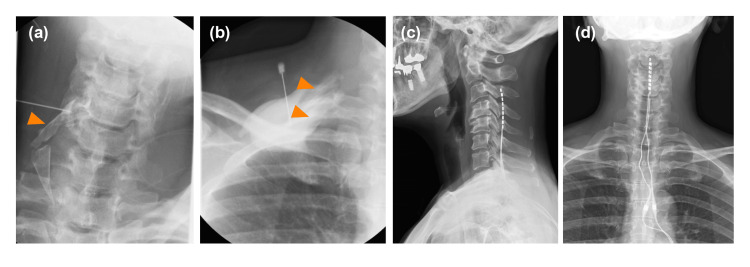
Peripheral nerve block and spinal cord stimulation. (a) C6 pulsed radiofrequency: The arrow indicates the contrasted right sixth cervical nerve root. (b) Brachial plexus block: The arrows indicate the contrasted anterior and middle scalene muscles. (c) Lateral radiographic image of spinal cord electrodes. (d) Frontal radiographic image of spinal cord electrodes.

She was admitted to our institution with persistent sharp electrical pain in her right first and second upper digits. According to the complex regional pain syndrome (CRPS) diagnostic criteria by the International Association for the Study of Pain, she did not have skin temperature or color changes, abnormal sweating, edema, range of motion or strength limitations, and alterations in hair, skin, or nail growth, which did not lead to a diagnosis of CRPS. The pain interrupted her dominant arm function and decreased her quality of life (QOL), which was assessed using the five-level EuroQol-5 dimensions with a maximum and minimum numerical rating scale (NRS) score of 7 and 5, respectively (Table 1). The pain impeded her from writing or using chopsticks. Since the excessive opioid dosage was ineffective, it was slightly reduced to avoid dependence. Although she experienced slight withdrawal symptoms, including anxiety or irritability, during the early phase of dosage reduction, they improved within several days. However, the dosage reduction aggravated the pain; therefore, a combination regimen of rehabilitation that consisted of isometric gripping and isotonic upper limb exercise, continuous epidural block with 0.8% mepivacaine at 2 ml/h, and peripheral nerve blocks, including brachial plexus block and cervical root block, was started (Figure 2a, 2b). Since these blocks effectively improved neuropathic pain, the opioid dosage could be reduced to 15 oral morphine mg equivalents per day for approximately one month. We performed a SCS percutaneous lead trial at a minimum opioid dosage. Two electrode leads (Avista™ MRI 8 contact lead; Boston Scientific Inc., Marlborough, Massachusetts, United States) were implanted in the cervical epidural space. The tips of the two spinal cord electrical stimulation electrodes were placed at the middle vertebral body of the C3 epidural space under the guidance of digital subtraction angiography (Figure 2c, 2d). The stimulation provided coverage of the painful region. In a seven-day trial period, SCS was set to deliver contour and tonic waveforms; moreover, the opioid treatment was completely stopped. Her NRS score gradually improved without opioid treatment; therefore, we performed permanent implantation (WaveWriter Alpha™ 16; Boston Scientific Inc.). Her NRS score remained stable without opioid treatment at six post-implantation months (Table 1). Furthermore, SCS analgesia improved her QOL, pain catastrophizing scale score, and function of the dominant arm. Moreover, it ameliorated the hyperalgesia, and she could write as well as use chopsticks with her right hand.

**Table 1:** Patient statement before and after spinal cord stimulation. SCS: spinal cord stimulation; NRS: numerical rating scale; PCS: pain catastrophizing scale; PSEQ: pain self-efficacy questionnaire; EQ-5D5L: five-level EuroQol-5 dimensions; HADS: Hospital and Anxiety Depression Scale

	NRS	PCS	PSEQ	EQ-5D5L	HADS	
					Anxiety	Depression
Pre-SCS	7	29	41	0.555	12	12
Post-SCS	3	10	34	0.624	8	5

## Discussion

This study had two considerations. First, a combination regimen of nerve blocks and rehabilitation during the dosage-tapering period may prevent aggravation of the pain scores. Second, peripheral median nerve injury resulting from a difficult venipuncture may cause chronic neuropathic pain, which may be alleviated by SCS.

There are several treatments for neuropathic pain, including nerve blocks, pharmacology, and exercise. According to the Neuropathic Pain Special Interest Group recommendations, there is limited evidence regarding interventional treatments for chronic peripheral neuropathic pain due to a lack of randomized controlled trials. However, case reports have indicated that nerve blocks can facilitate relief of chronic neuropathic pain. Although opioid agonists can be used to treat refractory neuropathic pain, they can aggravate hyperalgesia or tolerance [1]. In our case, we had to reduce the opioid dosage to eliminate the effect of opioid-induced hyperalgesia and tolerance, with nerve blocks being conducted to provide an analgesic effect. Neuraxial or peripheral blocks have an opioid-sparing effect during the perioperative period; however, it remains unclear whether these blocks have an opioid-sparing effect in patients with chronic neuropathic pain. Nonetheless, these blocks helped reduce opioid intake to a minimum dosage in our patient. This suggests that a regional block may be an alternative treatment during the dosage-tapering period. In a laboratory study, as pulsed radiofrequency enhances opioid analgesia in neuropathic pain, cervical root pulsed radiofrequency might have some opioid-sparing effect [2]. Rehabilitation exercise improves function rather than relieves pain; however, pain impedes physical exercise. Accordingly, interventional regional blocks allow patients to perform exercises without difficulty. Combined therapy is more effective than a single-modality approach. There is no strong evidence of what exercise protocol is the best for neuropathic pain, so a tailored program is needed for individuals. In pain-free patients, resistance exercise leads to exercise-induced hypoalgesia; however, pain exacerbation with exercise may occur in chronic pain patients [3]. We conducted resistance exercises which consisted of isometric gripping and isotonic upper limb exercises for our patient under regional block conditions. These exercises could alleviate the dominant arm function. The exercise-induced hypoalgesia with regional blocks may be one of the best treatments for chronic neuropathic pain.

In our patient, tonic and contour SCS provided sufficient analgesia without any hyperalgesia. SCS is known to relieve peripheral neuropathic pain, especially in patients with failed back syndrome and CRPS. However, previous reports have demonstrated the utility of high-frequency paresthesia-free, rather than tonic, SCS for peripheral nerve injury [4] since tonic stimulation induced paresthesia, which caused discomfort. Additionally, tonic SCS can aggravate neuropathic pain. Contrastingly, a laboratory study demonstrated that tonic SCS alleviated neuropathic pain caused by chronic peripheral nerve injury [5]. Peripheral nerve injury causes the microglial activation of the dorsal root ganglion (DRG) [6], which in turn releases colony-stimulating factor 1 (CSF-1); however, tonic SCS inhibits microglia to decrease spinal CSF-1 levels, leading to decreased neuroinflammation [5]. Our patient received two types of waveforms: tonic and contour. Contour stimulation, which is paresthesia-free, worked not to disturb her rest at night. Tonic stimulation of the painful area during the day decreased hyperalgesia. Therefore, tonic stimulation may have beneficial effects on neuropathic pain with hyperalgesia. Peripheral neuromodulation such as peripheral nerve stimulation (PNS) is another procedure to treat chronic neuropathic pain. Spinal cord stimulation was chosen because PNS lacks high-quality evidence and guidelines for intractable peripheral neuropathic pain and implantable versions are not approved in Japan.

## Conclusions

SCS relieved chronic persistent neuropathic pain and eliminated the need for opioid agonists. Concomitant nerve block treatment during the opioid-tapering period should contribute to a reduction in NRS score without interfering with rehabilitation. In case standard therapy is insufficient for peripheral nerve injury, SCS may positively affect refractory pain and QOL.
